# Computational analysis and prediction of PE_PGRS proteins using machine learning

**DOI:** 10.1016/j.csbj.2022.01.019

**Published:** 2022-01-22

**Authors:** Fuyi Li, Xudong Guo, Dongxu Xiang, Miranda E. Pitt, Arnold Bainomugisa, Lachlan J.M. Coin

**Affiliations:** a Department of Microbiology and Immunology, The Peter Doherty Institute for Infection and Immunity, The University of Melbourne, 792 Elizabeth Street, Melbourne, VIC 3000, Australia; b School of Information Engineering, Ningxia University, Yinchuan, Ningxia 750021, China; c Faculty of Engineering and Information Technology, The University of Melbourne, VIC 3000, Australia; d Queensland Mycobacterium Reference Laboratory, Brisbane, Australia

**Keywords:** PE_PGRS, Bioinformatics, Sequence analysis, Machine learning, Mycobacterial

## Abstract

*Mycobacterium tuberculosis* genome comprises approximately 10% of two families of poorly characterised genes due to their high GC content and highly repetitive nature. The largest sub-group, the proline-glutamic acid polymorphic guanine-cytosine-rich sequence (PE_PGRS) family, is thought to be involved in host response and disease pathogenicity. Due to their high genetic variability and complexity of analysis, they are typically disregarded for further research in genomic studies. There are currently limited online resources and homology computational tools that can identify and analyse PE_PGRS proteins. In addition, they are computational-intensive and time-consuming, and lack sensitivity. Therefore, computational methods that can rapidly and accurately identify PE_PGRS proteins are valuable to facilitate the functional elucidation of the PE_PGRS family proteins. In this study, we developed the first machine learning-based bioinformatics approach, termed PEPPER, to allow users to identify PE_PGRS proteins rapidly and accurately. PEPPER was built upon a comprehensive evaluation of 13 popular machine learning algorithms with various sequence and physicochemical features. Empirical studies demonstrated that PEPPER achieved significantly better performance than alignment-based approaches, BLASTP and PHMMER, in both prediction accuracy and speed. PEPPER is anticipated to facilitate community-wide efforts to conduct high-throughput identification and analysis of PE_PGRS proteins.

## Highlights

• PEPPER is the first machine learning-based predictor for PE_PGRS proteins.
• PEPPER is based on lightGBM and various sequence and physicochemical features.
• PEPPER can identify PE_PGRS proteins rapidly and accurately.
• The webserver of PEPPER and stand-alone tool are publicly available at http://web.unimelb-bioinfortools.cloud.edu.au/PEPPER/.

## Graphical abstract

**Figure:**
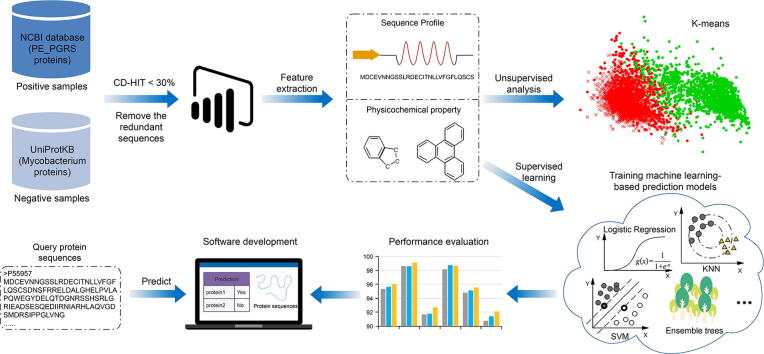

## 1 Introduction

*Mycobacterium tuberculosis* (MTB), the causative agent of pulmonary tuberculosis, infects one-third of the world’s population [1]. The emergence of multidrug-resistant and extensively drug-resistant strains, alarmingly rising numbers of patients with a coinfection of HIV and tuberculosis and variable efficacy of immunization with *Mycobacterium bovis* bacillus Calmette-Guerin have stressed the urgency of developing novel therapeutic intervention strategies for tuberculosis [2]. Decipher of the *Mycobacterium tuberculosis* H37Rv genome revealed approximately 10% of coding capacity to be accounted for two unrelated gene families encoding proline-glutamate (PE) and proline-proline-glutamate (PPE) gene family members, exemplified by the presence of PE and PPE motifs near the N-terminus of their gene products [3]. PE proteins are divided into three subfamilies: PE-only (less than100 amino acids in length); PE_unique, which present downstream of the PE domain a unique amino acid sequence of variable sequence; and PE_PGRS, which contain the polymorphic glycine-rich domain of variable sequence and size [4]. The MTB genome contains 65 PE_PGRS genes, although only 51 of these express a functional protein, at least in H37Rv [5]. These genes are found in all members of the MTB complex and a few other mycobacterial species as *Mycobacterium* marinum (∼148 genes) and *Mycobacterium* ulcerans (∼121 genes). However, PE_PGRS genes in these species show significant differences with those found in the complex [5].

It is widely considered that PE_PGRS proteins are involved in disease pathogenicity and progression, but their exact function remains elusive [6], [7], [8]. Some PE_PGRS proteins seem to be potential *Mycobacterium tuberculosis* candidate effectors, such as the PE_PGRS62 protein, which has been experimentally validated to have a role in virulence [9]. In addition, PE_PGRS proteins are proposed as molecular mantra to deflect host immunity [10], and are associated with the mycobacterial cell wall, influence cellular structure, and form mycobacterial colonies [8]. Furthermore, these proteins facilitate cell-surface interactions among mycobacteria and interactions with host macrophages [6]. More importantly, many previous studies have shown that the mycobacterial PE_PGRS proteins play essential roles in evading or modulation of the host immune system [11], [12]. Therefore, it is highly important to identify PE_PGRS proteins and elucidate their functional roles.

Due to the high GC content (approx. 80%), highly repetitive and a major source of polymorphism in the *Mycobacterium tuberculosis* complex, most genomic studies exclude these proteins, which results in poor understanding of these proteins [13]. Sequence identification and characterization by sequence search through databases is one of the primary ways of studying such variable proteins [14]. Therefore, alignment-based approaches, such as BLAST [15] and HMMER [16], and protein remote homology detection tools based on machine learning and BLAST, such as HITS-PR-HHblits [17], HHsuite [18], ProtDec-BLSTM [19], and ProtDet-CCH [20], can be used to identify PE_PGRS proteins. However, two major issues in these methods need to be addressed: (i) Both alignment-based approaches and protein remote homology detection methods require considerable computational resources and time, which are not suitable to perform high-throughput prediction and analysis of PE_PGRS proteins. (ii) Alignment-based approaches only consider the sequence information of the queried proteins. Their performance mainly depends on the quality and coverage of the search library. They are usually performed worse, especially for those proteins with low sequence similarity with the proteins in the search library. Machine learning combined with extensive sequence feature engineering techniques has been successfully used in many bioinformatics topics [[21], [22], [23], [24], [25], [26], [27], [28], [29], [30], [31], [32], [69]], and provide an alternative efficient and accurate strategy to study these enigmatic proteins. As such, we are highly motivated to leverage cutting-edge machine learning techniques to develop computational approaches to identify the PE_PGRS proteins rapidly and accurately.

In this study, we developed PEPPER (**PE**_PGRS **P**rotein **P**r**E**dicto**R**) based on machine learning techniques to identify PE_PGRS proteins. Firstly, we constructed a benchmark dataset by extracting manually annotated PE_PGRS proteins from NCBI and Swiss-Prot [33] databases. Then, we have comprehensively evaluated and compared 13 popular machine learning algorithms combined with a variety of sequence and physicochemical property features. PEPPER was developed based on the optimal predictor selected through extensive cross-validation and independent tests and further improved through feature selection. Empirical study results illustrated that PEPPER could achieve the significantly better predictive performance of PE_PGRS proteins and less computational time than BLASTP and PHMMER. In addition, we demonstrated the capacity of PEPPER by two case study proteins and applied PEPPER to conduct a proteome-wide prediction of PE_PGRS proteins. To the best of our knowledge, PEPPER is the first machine learning-based predictor for PE_PGRS proteins. We anticipate it will be widely applied to help discover and analyse novel PE_PGRS proteins and elucidate their functions.

## 2 Materials and methods

### 2.1 Overall framework of PEPPER

**Fig. 1:**
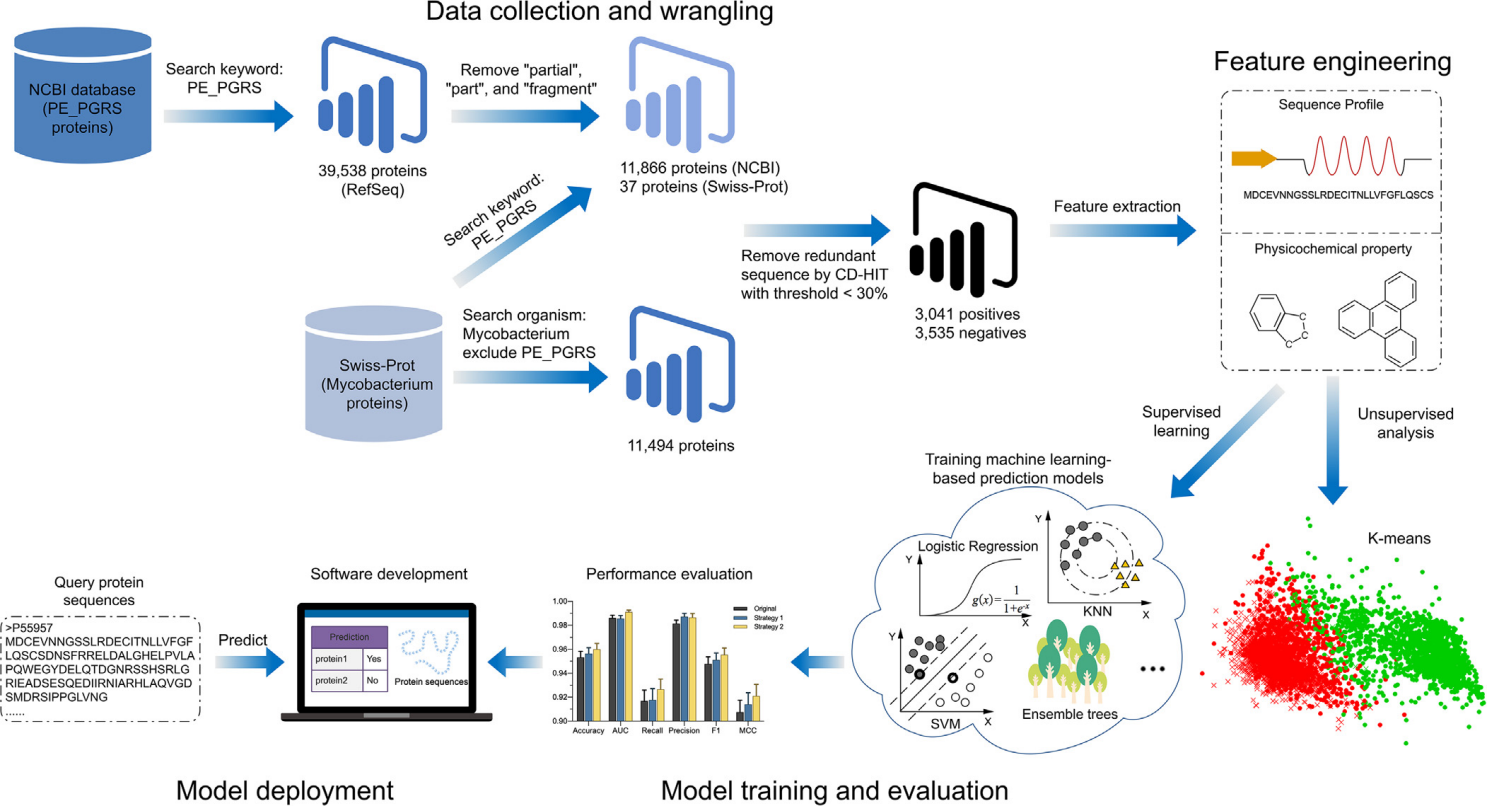
The overall framework of PEPPER.

Fig. 1 provides an overview of the design and performance evaluation process of PEPPER. Four major steps are involved in the construction and assessment of PEPPER, including data collection and wrangling, feature engineering, model training and evaluation, and model deployment. The first step is to collect the benchmark training and independent test datasets from publicly available NCBI and UniProt/SwissProt databases. In the second step, multi-faceted protein sequence profile and amino acid physicochemical property features are calculated and used as input for the machine learning algorithms. In the third step, 13 popular machine learning algorithms are employed to construct and explore the optimal predictors. In addition, feature selections are adopted to optimise the predictor. In the final step, an online webserver and a local stand-alone software are implemented for the model deployment.

### 2.2 Data collection

NCBI and UniProt/SwissProt are two popular databases that provide the scientific community with comprehensive, high-quality and freely accessible protein sequence resources. Firstly, we use the keyword “PE-PGRS” to search the NCBI Protein and Swiss-Prot database databases. For the NCBI database, there are 39,538 proteins obtained from RefSeq [34], then after removing those proteins annotated with “partial”, “part”, “fragment”, “PREDICTED”, “MODEL”, and “INFERRED”, 11,866 proteins are extracted. For the Swiss-Prot database, we searched protein names containing the keyword “PE-PGRS” and extracted 37 manually reviewed proteins. Those 11,903 PE-PGRS family proteins are used as the candidate positive samples. For candidate negative samples, we use the organism “*Mycobacterium*” to search the SwissProt database. By excluding the 37 PE-PGRS proteins, we have 11,494 candidate negative samples. To develop a reliable predictor and evaluate the model objectively, the PSI-CD-HIT program [35] with a strict sequence identity (SI) threshold of 30% [36], [37], [38], [39], [40], [41], [23] between any two protein sequences is used to discard highly-homologous sequences from the candidate positive and negative samples. Furthermore, the PSI-CD-HIT is also applied to remove the redundant sequence between positive and negative datasets at the SI threshold of 30%. Subsequently, we have 3,041 positive and 3,535 negative samples used for model training and testing. We randomly selected 70% of samples as the training dataset and the other 30% of samples as the independent test dataset. A statistical summary of training and independent test datasets is provided in Table 1.

**Table 1:** The statistical summary of the benchmark dataset used for training and testing.

	Original	After CD-HIT (<30%)	Training	Independent test
Positive	11,866	3041	2139	902
Negative	11,529	3535	2464	1071

### 2.3 Feature engineering

In this study, we used three groups of sequence-based features to encode the PE_PGRS protein sequences. Group 1 is amino acid composition features, Group 2 is Composition/Transition/Distribution (CTD) features, and Group 3 is the Conjoint Triad features. These three groups of feature encoding schemes are introduced in the following sections.

#### 2.3.1 Group 1. Amino acid composition features

Proteins with different amino acid sequences correspond to different structures, which result in differing functions. In this group, we consider four types of features, including Amino Acid Composition (AAC), Grouped Amino Acid Composition (GAAC), Composition of K-Spaced Amino Acid Pairs (CKSAAP) and Composition of K-Spaced Amino Acid Group Pairs (CKSAAGP).

##### 2.3.1.1 Amino Acid Composition (AAC)

The Amino Acid Composition encoding considers the frequencies of amino acids in the protein sequences. AAC calculates the frequencies of 20 natural amino acids as:

(1) $$AAC\left(a\right)=\frac{N(a)}{\mathrm{len}(P)}$$

where N(a) is the number of amino acid type a in the given protein sequence P, while len(P) is the length of the protein sequence P. Therefore, each protein sequence is represented as 20 AAC features [42], [43], [44], [45].

##### 2.3.1.2 Grouped Amino Acid Composition (GAAC)

Different amino acids have different physicochemical properties, such as hydrophobicity and molecular size. The Grouped Amino Acid Composition encoding classified the 20 natural amino acids into five groups according to their different physicochemical properties [46]. The five different amino acid groups are shown in Table S1. GAAC encodes protein sequences according to the frequency of each amino acid group, which is calculated as:

(2) $$PAAC\left(g\right)=\frac{N(g)}{\mathrm{len}(P)},g\in \left(g1,g2,g3,g4,g5\right)$$

(3) $$N\left(g_{t}\right)=\sum N\left(t\right),t\in g$$

where N(g) is the number of amino acids belonging to group g in the given protein P, len(P) is the length of the given protein P, and $N\left(t\right)$ is the number of amino acid type t.

##### 2.3.1.3 Composition of K-Spaced Amino Acid Pairs (CKSAAP)

CKSAAP is an encoding scheme that considers the frequency of amino acid pairs separated by k residues. The CKSAAP encodes a given protein sequence as a 400-dimensional feature vector, because 20 types of natural amino acids have 400 distinct types of k-spaced amino acid pairs (i.e., A(X∗k)A, A(X∗k)C, A(X∗k)D, …, Y(X∗k)Y). The A(X∗k)A means the amino acid pair AA separated by k residues, X implies any kind of amino acid and X∗k means k any residues. The CKSAAP feature vector is defined as:

(4) $${CKSAAP\left(P\right)}_{k}={\left(\frac{N_{A(X*k)A}}{N_{k}},\frac{N_{A(X*k)C}}{N_{k}},\frac{N_{A(X*k)D}}{N_{k}},\cdots ,\frac{N_{Y(X*k)Y}}{N_{k}}\right)}_{400}$$

(5) $$N_{k}=len\left(P\right)-k$$

where the value of each element in the feature vector represents the composition of the corresponding k-spaced residue pair in the given protein sequence. $N_{A(X*k)A}$ is the number of times k-spaced residue pair A(X∗k)A appears in the given protein P. $N_{k}$ is the total number of k-spaced residue pairs in the given protein P. In this study, we calculate the CKSAAP features with k=0,1,2,3,4,5 and the total number of features CKSAAP features is 400 × 6 = 2400.

##### 2.3.1.4 Composition of K-Spaced Amino Acid Group Pairs (CKSAAGP)

Similar to GAAC, the CKSAAGP encoding also classified the 20 natural amino acids into five groups according to their different physicochemical properties. Therefore, the CKSAAGP calculates the frequency of amino acid group pairs separated by any k residues. There are 25 k-spaced amino acid group pairs (i.e., $g1\left(X*k\right)g1$, $g1\left(X*k\right)g2$, $g1\left(X*k\right)g3$, …, $g5\left(X*k\right)g5$). The $g1\left(X*k\right)g1$ means the amino acid group pair g1g1 separated by k residues, X implies any kind of amino acid and X∗k means k any residues. The CKSAAGP feature vector is defined as:

(6) $${CKSAAGP\left(P\right)}_{k}={\left(\frac{N_{g1\left(X*k\right)g1}}{N_{k}},\frac{N_{g1\left(X*k\right)g2}}{N_{k}},\frac{N_{g1\left(X*k\right)g3}}{N_{k}},\cdots ,\frac{N_{g5\left(X*k\right)g5}}{N_{k}}\right)}_{25}$$

(7) $$N_{k}=len\left(P\right)-k$$

where the value of each element in the feature vector represents the composition of the corresponding k-spaced residue group pair in the given protein sequence P. $N_{g1\left(X*k\right)g1}$ is the number of times k-spaced residue pair $g1\left(X*k\right)g1$ appears in protein P. $N_{k}$ is the total number of k-spaced residue pairs in the given protein P. In this study, we calculate the CKSAAGP features with k=0,1,2,3,4,5, and the total number of CKSAAP features is 25 × 6 = 150.

#### 2.3.2 Group 2. Composition/Transition/Distribution (CTD) feature

The Composition Transition and Distribution (CTD) feature is a type of physicochemical property of amino acid sequences, representing the global distribution patterns and physicochemical properties of the protein sequences [47], [48]. CTD feature includes composition in CTD (CTDC), transition in CTD (CTDT) and distribution in CTD (CTDD). There are 13 types of physicochemical properties in the CTD encoding scheme, including seven types of hydrophobicity (e.g., PRAM900101, ARGP820101, ZIMJ680101, PONP930101, CASG920101, ENGD860101, and FASG890101), normalised van der Waals volume, polarity, polarizability, charge, secondary structure, and solvent accessibility. According to their attributes, 20 natural amino acids are categorised into three groups for each property. Supplementary Table S2 provides the three groups information of 20 natural amino acids. For example, a 20-amino acid example sequence “RKEDQNGASTPHYCLVIMFW” will be encoded as “11111122222223333333” according to hydrophobicity_PRAM900101 group in Supplementary Table S2, where “1”, “2”, “3” means “Group1”, “Group2”, “Group3”, respectively.

In this study, **CTDC** is defined as a 13 × 3 = 39-dimensional feature vector as follows:

(8) $$CTDC\left(P\right)={\left(\frac{N_{P1\_G1}}{\mathrm{len}(P)},\frac{N_{P1\_G2}}{\mathrm{len}(P)},\frac{N_{P1\_G3}}{\mathrm{len}(P)},\cdots ,\frac{N_{P13\_G1}}{\mathrm{len}(P)},\frac{N_{P13\_G2}}{\mathrm{len}(P)},\frac{N_{P13\_G3}}{\mathrm{len}(P)}\right)}_{39}$$

where len(P) means the sequence length of given protein P; $N_{P1\_G1}$ means, in given protein P, the number of amino acids belongs to the Group 1 (G1) of property 1 (G1, which is hydrophobicity_PRAM900101 according to Supplementary Table S1). Similarly, $N_{P13\_G3}$ means the number of amino acids belongs to Group 3 of property 13 (Solvent Accessibility) in given protein P. For the example sequence, len(P)=20, and for property 1 (P1= hydrophobicity_PRAM900101), $N_{P1\_G1}=6$, $N_{P1\_G1}=7$, and $N_{P1\_G1}=7$, because 6 amino acids belong to Group 1, 7 amino acids belong to Group 2, and 7 amino acids belong to Group 3. Therefore, the composition features of hydrophobicity_PRAM900101 are calculated as 6/20, 7/20, and 7/20, respectively. The CTDC features for other 12 properties can be calculated in a similar way.

**CTDT** calculates the frequency of a Group 1 residue followed by a Group 2 residue or vice versa. For example, a CTDT (transition) from Group 1 to Group 2 is the percentage frequency with which a Group 1 residue is followed by a Group 2 residue or a Group 2 residue by a Group 1 residue. The CTDT features can be calculated as:

(9) $$CTDT\left(P\right)={\left(\frac{T_{P1\_12}}{N_{T}},\frac{T_{P1\_13}}{N_{T}},\frac{T_{P1\_23}}{N_{T}},\cdots ,\frac{T_{P13\_12}}{N_{T}},\frac{T_{P13\_13}}{N_{T}},\frac{T_{P13\_23}}{N_{T}}\right)}_{39}$$

(10) $$T_{Pi\_MN}=N_{\mathrm{Pi}}\left(MN\right)+N_{\mathrm{Pi}}\left(NM\right)$$

(11) $$N_{T}=len\left(P\right)-1$$

where len(P) is the sequence length of given protein P, $T_{Pi\_MN}$ is the transition from Group M to Group N of property i. For the given example sequence, $N_{T}=19$, and for property 1 (P1= hydrophobicity_PRAM900101), $T_{P1\_12}=N_{P1}\left(12\right)+N_{P1}\left(21\right)=1$, where $N_{P1}\left(12\right)$ and $N_{P1}\left(21\right)$ are the numbers of dipeptide encoded as “12” and “21” in the sequence, respectively. Therefore, $T_{P1\_12}=1/19$, $T_{P1\_13}=0/19$, and $T_{P1\_23}=1/19$. The CTDT features for other 12 properties can be calculated in a similar way. Accordingly, CTDT is also presented as a 13 × 3 = 39-dimensional feature vector.

**CTDD** describes the distribution of each physicochemical property in the sequence [47]. It calculates five distribution features for each physicochemical group according to the five sequence lengths (in percent), within which the first, 25%, 50%, 75%, and 100% of the amino acids with a certain property are contained. For the property 1 of (P1= hydrophobicity_PRAM900101) given example sequence, there are 6 amino acids (‘RKEDQN’) in group 1. The first residue of the given sequence to group 1, hence the first feature is calculated as (1/20) × 100%=5. Twenty-five percent of group 1 amino acids (25%×6≈2 amino acids) are contained within the first two residues. Therefore the second feature is calculated as (2/20) × 100%=10. Similarly, 50% of group 1 amino acids (50%×6 = 3) are within the first three residues of the example sequence. Therefore, the third feature is calculated as (3/20) × 100%=15. Then, 75% of group 1 amino acids (75%×6 = 4.5≈5) are within the first five residues of the example sequence. Therefore, the fourth and fifth features are calculated as (5/20) × 100%=25 and (6/20) × 100%=30, respectively. Similar calculations were performed for groups 2 and 3. For example, there are 7 amino acids (‘GASTPHY’) in group 2. The first group 1 amino acid is located at the 7th residue of the sequence. Therefore the first feature of group 2 is calculated as (7/20) × 100%=35. Twenty-five percent of group 2 amino acids (25%×7≈2 amino acids) are contained with the first eight residues (positions 7 and 8). Hence the second feature of group 2 is calculated as (8/20) × 100%=40. Others can be calculated in a similar method. Therefore, CTDD is presented as a 13 × 3 × 5 = 195-dimensional feature vector.

#### 2.3.3 Group 3. Conjoint Triad feature

Group 3 contains two types of feature encoding schemes, i.e., Conjoint Triad, and K-Spaced Conjoint Triad. **Conjoint Triad (CTriad)** feature describes the properties of a triad amino acid unit, which is a combination of any three amino acids [49]. CTriad classifies the 20 nature amino acids into seven groups and uses these for the feature encoding, which include [A, G, V] for group 1, [I, L, F, P] for group 2, [Y, M, T, S] for group 3, [H, N, Q, W] for group 4, [R, K] for group5, [D, E] for group 6, and C for group 7. The CTriad feature is defined as:

(12) $$Conjoint\_Triad\left(P\right)={\left(\frac{f_{1}-min}{\max},\frac{f_{2}-min}{\max},\cdots ,\frac{f_{342}-min}{\max},\frac{f_{343}-min}{\max}\right)}_{343}$$

(13) $$f_{i}=N_{\mathrm{Vi}}i\in \left(1,2,3,\cdots ,343\right)$$

where $N_{\mathrm{Vi}}$ denotes the number of type Vi appearing in the given protein sequence P and Vi represents triad type, containing three contiguous amino acids. The $\max=max(f_{1},f_{2},\cdots ,f_{343})$ and $\min=min(f_{1},f_{2},\cdots ,f_{343})$. Thus, the longer protein sequences are more likely to have larger $f_{i}$ values. To eliminate the factor of protein length, the feature is normalised, and the feature vector is 7^3^ = 343-dimension.

**The K-Spaced Conjoint Triad** (KSCTriad) is based on CTriad, besides the numbers of three continuous amino acid units, KSCTriad also considers the continuous amino acid units separated by any k residues.

### 2.4 Machine learning algorithms

The PE_PGRS protein prediction task is a binary classification problem, e.g., classifying PE_PGRS proteins with non-PE_PGRS proteins. To find the optimal machine learning algorithms for PE_PGRS protein prediction, this study comprehensively evaluate and compare 13 popular supervised machine learning algorithms for PE_PGRS protein prediction, including CatBoost [50], extreme gradient boosting (XGBoost) [51], Light Gradient Boosting Machine (lightGBM) [52], Gradient Boosting Decision Tree (GBDT) [53], Adaptive Boosting (AdaBoost) [54], Random Forest (RF), Extra Trees, Logistic Regression (LR), Decision Tree, Naïve Bayes (NB), Support Vector Machine (SVM), K-Nearest Neighbours classifier (KNN), and Linear Discriminant Analysis (LDA). These machine learning algorithms are successfully applied in many bioinformatics sequence-based prediction tasks [37], [38], [39], [41], [55], [56], [57], [58]. For LR, NB, SVM, KNN, and LDA, the feature set is first standardised by using Z-score normalisation. While for tree-based algorithms, e.g., CatBoost, XGBoost, lightGBM, GBDT, AdaBoost, RF and Extra Trees, the original features are used as they are not sensitive to the variance in the data. The hyper-parameters of each classifier are optimised by the Bayesian optimisation algorithm [59] and the performance comparison for these 13 algorithms is conducted on the training dataset with 10 times 10-fold cross-validation tests and report the average performances. After extensive performance evaluation, we finally selected lightGBM build the model of PEPPER. LightGBM is an effective extension of GBDT, an iterative decision tree algorithm, which learns a boosting model from mistake residual errors and performs prediction by adding the previous predictions of all trained trees. LightGBM has been proposed to improve efficiency and reduce calculation cost by employing a histogram algorithm [52]. Furthermore, lightGBM algorithm equips the Gradient-based One-Side Sampling (GOSS), Exclusive Feature Bundling (EFB), and Leaf-wise Tree Growth strategies to reduce computational complexity and improve the accuracy. In this study, lightGBM was implemented using the lightgbm package in Python (https://github.com/Microsoft/LightGBM).

### 2.5 Performance evaluation

The predictive performance of prediction models is compared and evaluated by several commonly used performance metrics [36], [37], [60], including Accuracy, Recall, Precision, F1, Matthew's Correlation Coefficient (MCC) and area under the receiver-operating curves (AUC). Accuracy, Recall, Precision, F1, and MCC are respectively defined as:

(14) $$Accuracy=\frac{\mathrm{TP}+TN}{\mathrm{TP}+TN+FP+FN}$$

(15) $$Recall=\frac{\mathrm{TP}}{\mathrm{TP}+FN}$$

(16) $$Precision=\frac{\mathrm{TP}}{\mathrm{TP}+FP}$$

(17) $$F1=\frac{2\times Precision\times Recall}{Precision+Recall}$$

(18) $$MCC=\frac{\mathrm{TP}\times TN-FP\times FN}{\sqrt{\left(TP+FP\right)\times \left(TP+FN\right)\times \left(TN+FP\right)\times \left(TN+FN\right)}}$$

where TP, TN, FP, and FN represent the number of true positives, true negatives, false positives, and false negatives, respectively.

## 3 Results and discussion

### 3.1 Sequence analysis

#### 3.1.1 Sequence length distribution and amino acid frequencies of PE_PGRS proteins

**Fig. 2:**
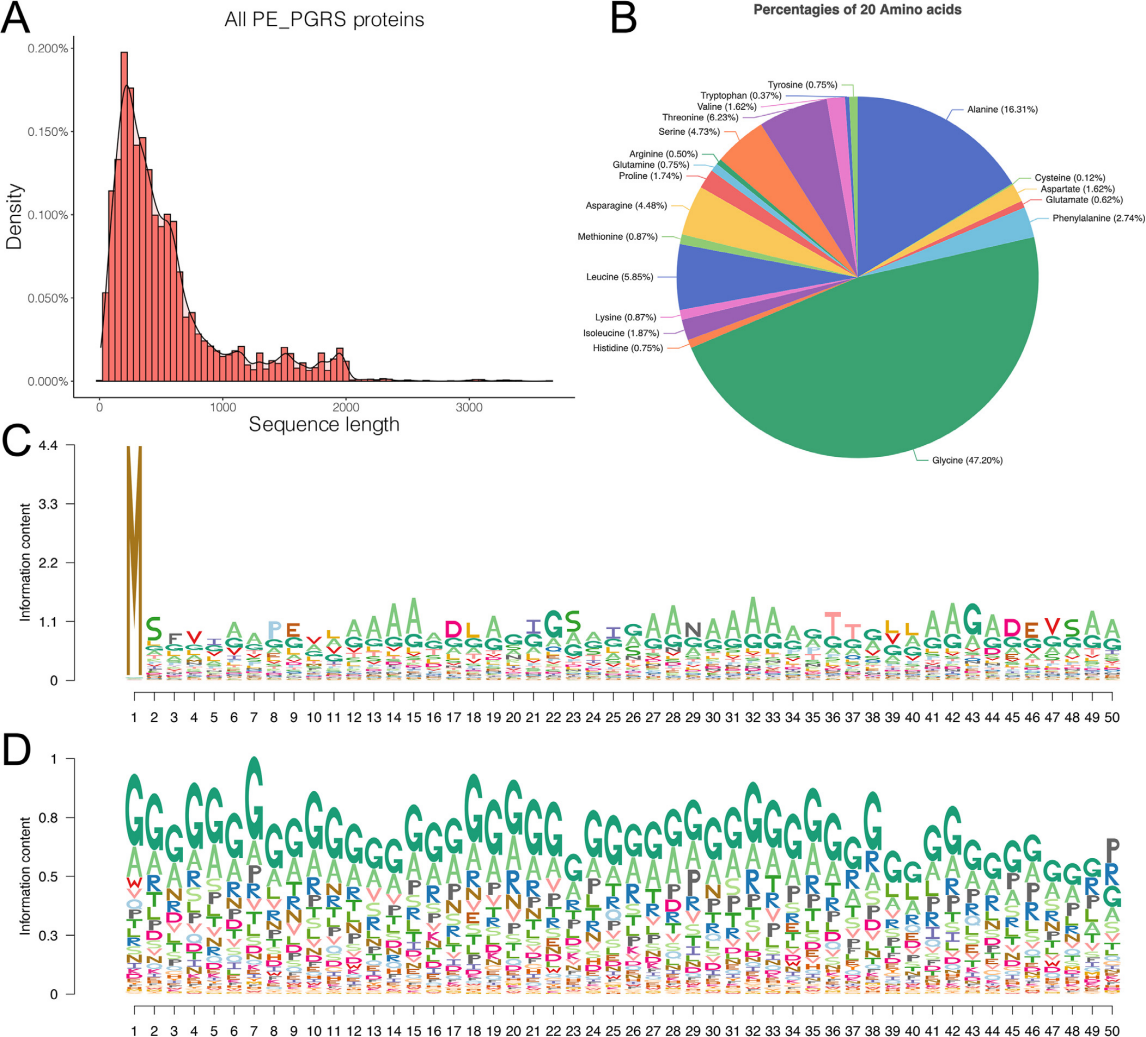
Sequence analysis of known PE_PGRS proteins. (**A**) Distribution of all collected PE_PGRS proteins according to their protein sequence lengths. (**B**) Frequency distributions of 20 amino acids in all accumulated PE_PGRS proteins. (**C**) Sequence-logo of the N-terminal sequence of PE_PGRS proteins. (**D**) Sequence-logo of the C-terminal sequence of PE_PGRS proteins.

In this section, we analysed the characteristic sequence lengths and amino acid frequencies of known PE_PGRS proteins using the collected dataset. We merged the training and independent test dataset to make a more comprehensive analysis. The variation of protein sequence length reflects the functional diversity and complexity of the protein family. To identify the sequence length distribution of PE_PGRS proteins, we calculated their protein-sequence lengths and summarised the results in Fig. 2**A**. The histogram shows that the proteins comprised of ∼ 200 amino acids have the most significant density, almost 0.2%. Of note, most PE_PGRS proteins have less than 2000 amino acids, and there are > 86.2% PE_PGRS proteins with length less than 1000. These results demonstrate the distribution of protein sequence length is relatively concentrated.

Besides, the distribution of the amino acid frequencies is also related to the evolution and function of proteins. Therefore, we analysed the frequency distributions of 20 natural amino acids in all PE_PGRS proteins. The percentage value of each amino acid of all PE_PGRS proteins is shown in the pie chart (Fig. 2**B**). It is apparent that glycine (G) is the most frequently occurring residue in the PE_PGRS proteins, accounting for 47.20% in all 20 amino acids. Glycine is one of the proteinogenic amino acids encoded by all the codons starting with GG (e.g., GGU, GGC, GGA, GGG). Due to its compact form, glycine is integral to the formation of alpha-helices in the protein secondary structures. This result is consistent with previous research that PE_PGRS proteins are glycine-rich proteins [61]. The second most abundant amino acid is alanine (A), which accounts for precisely 16.31% of the total and threonine (T) accounts for 6.23% ranked the third. It is also observed that leucine (L), serine (S), and asparagine (N) have similar percentages, which are 5.85%, 4.73%, and 4.48%, respectively, while the other 14 amino acids together account for ∼ 15% in total.

#### 3.1.2 Analysis of sequence motifs of known PE_PGRS proteins

Prior researches have reported that PE_PGRS proteins have special N-terminal and C-terminal domains [62], [63]. To better understand the N-terminal and C-terminal sequence profiles of PE_PGRS proteins, we examined the N- and C-terminal sequences of PE_PGRS proteins with the Logolas package [64], which is an R package to characterise and display the statistically significant sequence motifs. In this study, we employed a window size of 50 amino acids to extract the N- and C-terminal sequences from the curated PE_PGRS proteins, and these proteins with less than 50 amino acids were removed. The generated sequence logo diagrams for N- and C-terminal sequences are shown in Fig. 2**C** and Fig. 2**D**, respectively. At each position of the sequence logo plots, amino acids are stacked together, and the total height of the stack is related to the information content of the corresponding position. Furthermore, the height of each amino acid is proportional to its relative frequency, and the amino acids are ordered by their frequencies.

Several notable amino acid preferences in N-terminal sequences are observed in Fig. 2**C**. First, we can find glycine (G) has relatively higher frequencies than any other amino acids in every position of the N-terminal sequences except position 1, which is dominated by methionine, the initiation codon. Alanine (A) is the second most abundant amino acid in the N-terminal sequences, which is present across multiple positions, including positions 6, 7, 12–16, 19, 27, 28, 30–34, 41, 42, 44, 49, and 50. In addition, a tetra-peptide motif DEVS at the positions 45–48 and DXXS (X represents any amino acids) at the positions 44–47, which are consistent with the findings in previous research [62], [63]. These two motifs could have evolved for serine phosphorylation and caspase-3 binding recognition. The sequence logo in Fig. 2**D** shows that the C-terminal sequences of PE_PGRS proteins also exhibit an enrichment with glycine (G) and alanine (A) residues across all these positions. These results are consistent with the findings in Fig. 2**B**, that glycine (G) and alanine (A) are the top two most frequently occurring amino acids in PE_PGRS proteins. Besides, asparagine (N) and arginine (R) are also enriched in C-terminal sequences compared with other residues, although arginine (R) only accounts for 0.5% in total (Fig. 2**B**). These observations are consistent with studies that C-terminal sequences of PE_PGRS proteins bearing GGA or GGN multiple tandem repeat structure, and glycine (G) and alanine (A) are enriched in a GGAGGX motif [63], [65].

### 3.2 Unsupervised analysis

**Fig. 3:**
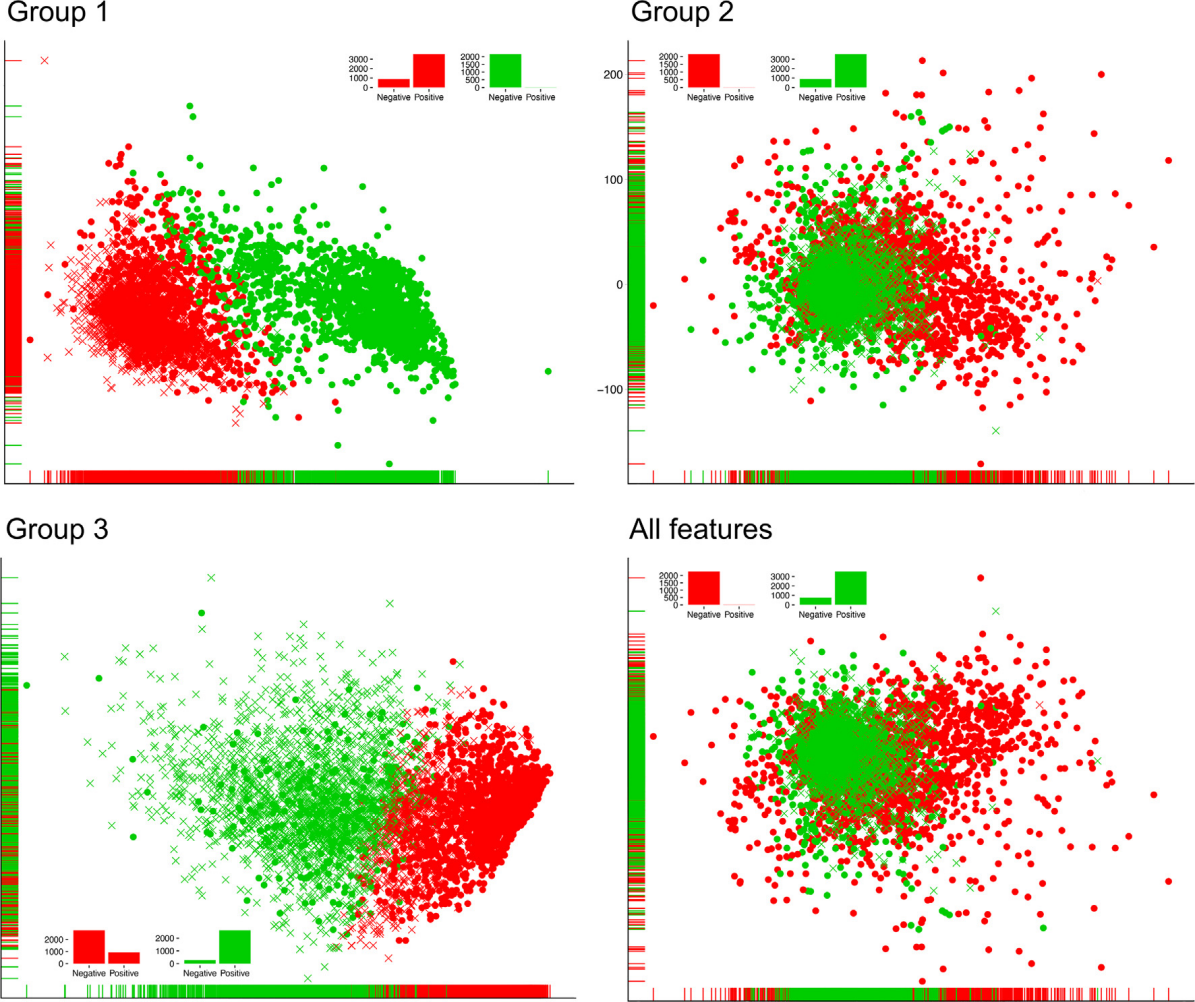
Distribution and clustering of PE_PGRS and non-PE_PGRS proteins based on three groups of features and all features. For each feature group, samples were clustered into two groups using the *K*-means algorithm, different clusters are represented by different colours. The PE_PGRS and non-PE_PGRS proteins are presented in different shapes, where dots mean PE_PGRS proteins and multiplication signs represent non-PE_PGRS proteins. The inset bar chart in each sub-figure shows the samples distribution (PE_PGRS vs. non-PE_PGRS) in each cluster.

We employed three groups of sequence and physiochemical features in this study to intuitively explore each feature group’s ability and all features to distinguish PE_PGRS proteins and non-PE_PGRS proteins. We conducted an unsupervised analysis by employing *K*-means algorithm [66] and the results are presented in Fig. 3. For each feature group, we employed *K*-means to conduct a two-class clustering on all positive (PE_PGRS) and negative (non-PE_PGRS) samples in training and independent test datasets. Besides, the samples were mapped onto the two-dimensional feature space by using the Principal Component Analysis (PCA) algorithm, which allows presenting the clusters with a two-dimensional scatter plot and measures the differences of samples by their mutual distances in space. As shown in Fig. 3, positive (PE_PGRS) and negative (non-PE_PGRS) samples are represented with different shapes, e.g., a dot means a positive sample, and a multiplication sign represents a negative sample. And the clustering results are represented by different colours (e.g., green and red). The inset bar chart in each sub-figure shows the distribution of positive and negative samples in each cluster (the detailed results are summarised in Supplementary Table S3**)**. Overall, we can see that the distribution of the positive and negative samples represented by any of the three groups of features, or All features is randomly scattered, and it is difficult to observe an apparent boundary between different classes on each sub-figure.

However, when further investigating the clustering results, we find that Group 1, Group 2, and All features achieved promising classification performance. For Group 1 and Group 2, positive samples are dominated in Cluster 2 (99.63% for Group 1 and 99.72% for Group 2) and Cluster 1 for All features (99.56%). These clustering results not only show a high division of positive and negative samples but also exhibit a low mixture rate of two classes within each cluster. For Group 1 and Group 2, positive samples are more likely to be classified into Cluster 1, and there are 80.03% and 79.91% negative samples in Cluster 1 of Group 1 and Group 2, respectively. For All features, 82.13% of samples in Cluster 2 are negative samples. Although Group 3 achieved relatively lower-division performance than the other two, it also showed promising classification ability (90.53% negative samples in Cluster 1 and 74.95% positive samples in Cluster 2).

The good performances of these three groups of features demonstrate that the sequence composition features, and physicochemical property encodings can provide a suitable characterisation of the differences between PE_PGRS/non-PE_PGRS proteins. In addition, we can find that All features achieved the best balance between the high division and low mixture rate of two classes, which provide a high-quality discriminative ability and a reliable feature space to build robust supervised prediction models. Therefore, we applied All features as the initial feature set to conduct the supervised learning analyses in the following sections.

### 3.3 Performance evaluation of supervised learning algorithms on the training dataset

This section conducted a comprehensive performance and computational time evaluation and compared 13 popular supervised machine learning algorithms using 10-fold cross-validation tests on the training dataset. As mentioned in Section 3.2, All features achieved the best predictive performance in terms of high division and low mixture rate of positive and negative samples. Therefore, we used All features as the initial feature set to train and evaluate the prediction models. The performance comparison was conducted on the training dataset with 10 times 10-fold cross-validation tests, and the average results are provided in Table 2.

**Table 2:** Performance comparison results of 13 popular machine learning algorithms on the training dataset (the classifiers are ranked according to the accuracy, and the best values are marked in bold).

Classifier	Accuracy	AUC	Recall	Precision	F1	MCC	TT (Sec)
CatBoost	**0.9559**	0.9850	0.9168	**0.9873**	**0.9506**	**0.9131**	286.863
XGBoost	0.9557	0.9848	**0.9186**	0.9849	0.9505	0.9125	19.777
lightGBM	0.9533	**0.9861**	0.9168	0.9814	0.9478	0.9076	8.471
GBDT	0.9520	0.9816	0.9154	0.9799	0.9464	0.9050	52.49
Random Forest	0.9511	0.9802	0.9116	0.9819	0.9453	0.9035	2.905
Extra Trees	0.9483	0.9804	0.9051	0.9822	0.9419	0.8981	4.696
AdaBoost	0.9420	0.9735	0.9182	0.9554	0.9362	0.8839	10.669
Logistic Regression	0.9333	0.9621	0.8953	0.9586	0.9256	0.8673	5.558
Decision Tree	0.9187	0.9182	0.9102	0.9152	0.9123	0.8373	2.951
Naïve Bayes	0.9057	0.9160	0.8621	0.9299	0.8945	0.8117	0.1
SVM	0.8657	0.8535	0.7118	0.9923	0.8289	0.7503	0.825
KNN	0.8553	0.8971	0.7274	0.9495	0.8232	0.7229	6.25
LDA	0.8040	0.8260	0.8139	0.7757	0.7942	0.6083	11.757

We can make several important observations from Table 2. First, tree-based classifiers achieved overall better predictive performance compared with other algorithms and the top seven classifiers in terms of accuracy were all tree-based algorithms. Second, we can find that CatBoost achieved the best performance in terms of accuracy, precision, F1 and MCC, while XGBoost secured the best recall and lightGBM was the best-performing classifier in terms of AUC. Third, in terms of the time-usage of model training, Naïve Bayes was the most time-saving algorithm for model training, and lightGBM was the most time-saving method among the four best gradient boosting tree algorithms. While CatBoost required considerable time for model training, which was ∼ 34 times longer than lightGBM. Finally, we selected the top five classifiers in terms of accuracy, including CatBoost, XGBoost, lightGBM, GBDT and RF, to do the further tests.

### 3.4 Performance evaluation and comparison with state-of-the-art alignment-based approaches and remote homology detection tools on the independent test dataset

**Fig. 4:**
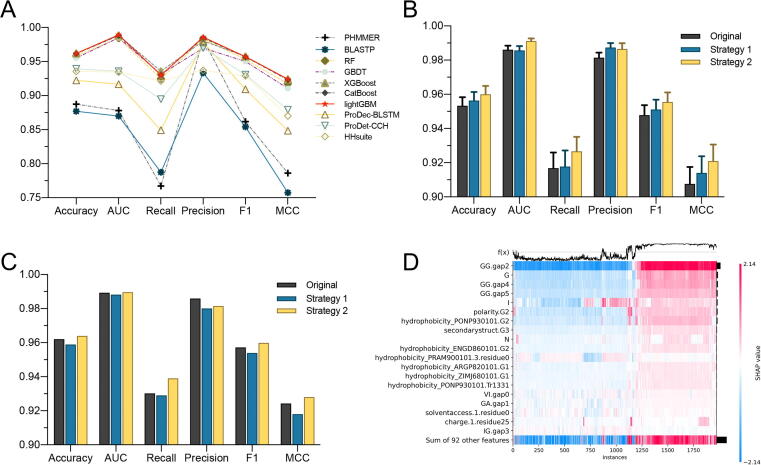
(**A**) Performance evaluation and comparison of top five machine learning-based predictors with BLASTP and PHMMER. (**B**) Performance comparison results of two feature selection strategies on the training dataset. (**C**) Performance comparison results of two feature selection strategies on the independent test dataset. (**D**) Heatmap plot of the SHAP values for the top 20 important features on the independent test dataset.

This section further evaluated and compared the predictive performance of the top 5 classifiers selected in Section 3.3 with two state-of-the-art alignment-based approaches, including BLASTP [15] and PHMMER [16], and three remote homology detection tools, including HHsuite [18], ProtDec-BLSTM [19], and ProtDet-CCH [20], based on the independent test dataset. The independent test dataset searched against the training dataset by alignment-based approaches and remote homology detection tools. For each protein sequence in the independent test dataset, the predicted label was assigned as the same top-matched protein label with the lowest E-value in the training dataset. For example, if a protein’s top matched protein belongs to the positive samples in the training dataset, we marked the predicted label as positive and vice versa. Therefore, we compared the top five machine learning-based predictors, including CatBoost, XGBoost, lightGBM, GBDT and RF, with two alignment-based approaches and three remote homology detection tools using the same independent test dataset. The performance comparison results are summarised in Fig. 4**A**, and the detailed results are provided in Table 3.

**Table 3:** Performance comparison results of top five machine learning models, two popular alignment-based approaches (BLASTP and PHMMER), and three remote homology detection tools (ProDec-BLSTM, ProDet-CCH, and HHsuite) on the testing dataset.

Algorithm	Accuracy	AUC	Recall	Precision	F1	MCC
lightGBM	**0.9620**	**0.9893**	0.9302	**0.9859**	0.9572	**0.9243**
XGBoost	**0.9620**	0.9884	**0.9357**	0.9803	**0.9575**	0.9240
CatBoost	0.9615	0.9882	0.9302	0.9847	0.9567	0.9233
GBDT	0.9554	0.9849	0.9313	0.9700	0.9502	0.9105
RF	0.9584	0.9845	0.9246	0.9835	0.9531	0.9173
BLASTP	0.8770	0.8699	0.7874	0.9333	0.8540	0.7573
PHMMER	0.8875	0.8780	0.7672	0.9830	0.8618	0.7861
ProDec-BLSTM	0.9225	0.9167	0.8492	0.9783	0.9092	0.8485
ProDet-CCH	0.9392	0.9357	0.8947	0.9700	0.9308	0.8790
HHsuite	0.9356	0.9345	0.9213	0.9369	0.9290	0.8702

We can find that, in general, the five machine learning models achieved the best performance, followed by the three remote homology detection tools, and two alignment-based approaches performed worst. More specifically, lightGBM and XGBoost achieved overall better predictive performance compared with others, where lightGBM achieved the best predictive performance in terms of accuracy, AUC, precision, and MCC, while XGBoost performed best in terms of recall and F1. In contrast, BLASTP and PHMMER performed worse than the machine learning algorithms, with the only exception that PHMMER achieved 0.9830 in terms of precision, which ranked 4th in these ten compared methods. However, the line chart in Fig. 4**A** demonstrates the machine learning predictors had an overwhelming advantage over alignment-based approaches and remote homology detection tools in terms of other performance evaluation metrics. The reason is that we employed both sequence profiles and physicochemical properties in training the machine learning predictors, while alignment-based approaches are only focused on sequence homology patterns. Therefore, machine learning-based predictors can explore more valuable patterns and enable more reliable prediction results.

Considering the results of both training and independent tests, we determined that CatBoost, XGBoost and lightGBM were the top three performed algorithms. CatBoost achieved overall better results on the training dataset, while lightGBM and XGBoost obtained better independent test results. Besides, lightGBM also earned the best AUC on the training dataset and was the most time-saving algorithm compared with XGBoost and CatBoost. Therefore, we finally selected to optimise lightGBM to develop our prediction tool PEPPER because lightGBM can make the prediction fast and accurately.

### 3.5 Feature selection further improved the performance

As mentioned before, we used three groups of features to train the machine learning models. However, it is likely that the initial feature sets probably have some redundant and noisy features, which have negative impacts on model training. Therefore, to further improve the predictive performance of lightGBM, we employed feature selection to identify the informative feature subsets. In this section, we compared two two-step feature selection strategies, Strategy 1 and Strategy 2, to find the optimal feature subset. Strategy 1 combines mRMR (minimum redundancy maximum relevance) [67] and incremental feature selection (IFS) algorithms, which is widely used in many bioinformatics tasks [38], [56], [57], [58]. In contrast, Strategy 2 combines feature importance of lightGBM and the IFS algorithm. The only difference between these two strategies is the first step, e.g., Strategy 1 ranked all the initial features according to the mRMR algorithm. However, we used the feature importance score calculated by lightGBM to rank the features in Strategy 2. Then, in the second step, the IFS algorithm combined with lightGBM to find the optimal feature subsets on the training dataset. For the ranked feature set $F=(f_{1},f_{2},\cdots f_{n-1},f_{n})$ resulted in the first step (n represents the number of features), IFS constructs n feature subsets by adding one feature by adding one feature from F. For example, the i-th feature subset is defined as $F_{i}=\left(f_{1},f_{2},\cdots f_{i}\right)$. Then, n lightGBM classifiers were trained by 10 times 10-fold cross-validation tests and the feature set $F_{i}$ that achieved the highest AUC was selected as the optimal feature set. The feature selection results of Strategy 1 and Strategy 2 compared with the original model on training and independent test dataset are shown in Fig. 4**B and 4C** (detailed results provided in Supplementary Table S4), respectively.

The results showed that Strategy 2 performed best in terms of accuracy, AUC, recall, F1 and MCC compared with Strategy 1 and the original model on both training and independent test datasets. In addition, the optimal feature subset selected by Strategy 2 only has 111 features, which significantly reduced the feature dimensional compared with the original feature set and the optimal feature set selected by Strategy 1. Therefore, the feature selection conducted by Strategy 2 further enhances the accuracy of the prediction model and reduces the computational complexity for model training. Finally, these 111 optimal features were used to train the lightGBM model and build our predictor, PEPPER, for PE_PGRS protein prediction. The learning curves of PEPPER with 10-fold cross-validation tests on the training dataset are provided in Supplementary Figure S1. From the learning curves we can find the training score is always around the maximum, and the validation score could be little increased with more training samples, but from a larger perspective, it is basically maintained in a stable range. The corresponding ROC curves and confusion matrix of PEPPER are provided in Supplementary Figure S2. The statistic summarises of the optimal feature subset are provided in Supplementary Table S5. We can find that there are 62 CKSAAP features in the optimal feature subset, which account for 2.58% features of all CKSAAP features. In addition, there are also 15 CTDD, 12 CTDC, 6 ACC and CKSAAGP, 5 CTDT and CTriad features in the optimal feature subset. Overall, CTDC and ACC features were more informative and more proportional features were selected as the optimal features. The selected CTDC features account for 30.77% (12 of 39) of all CTDC features, and 30% (6/20) of AAC features were selected in the optimal feature subset. In comparison, CTriad and CKSAAP were relatively sparse, as only 1.46% (5/343) and 2.58% (62/2400) of all CTriad and CKSAAP features were selected in the optimal feature subset.

### 3.6 Model interpretation

PEPPER trained on the optimal feature subset selected by the two-step feature selection achieved very competitive performance in predicting PE_PGRS proteins. However, the contribution and directionality of the optimal features for the lightGBM model are still unknown. Therefore, we conducted the model interpretation analyses by leveraging the Shapley Additive explanation (SHAP) algorithm [68] to identify the most contributed features and their relationships with the prediction results of PEPPER. The directionality of a feature means how a feature value relates to the prediction results of the predictor. Fig. 4**D** and Supplementary Figure S2 show the heatmap matrices of the top 20 critical features ranked based on the SHAP value for PEPPER on the independent test dataset and training dataset, respectively. In the heatmaps, the samples on the x-axis, the model's inputs on the y-axis, and the SHAP values are represented on a colour scale. The samples are ordered based on hierarchical clustering in SHAP by their explanation similarity; therefore, the samples with the same prediction results were grouped together, such as proteins with a high impact from the CKSAAP feature GG.gap2 shown in Fig. 4**D** and Supplementary Figure S3. The prediction results of PEPPER are shown in the line chart above the heatmap matrix (namely f(x)), the global importance of each feature is represented in the bar plot on the right-hand side of the heatmap, and the top 20 important features are sorted according to the global importance. In addition, we also plot the beeswarm plot of the top 20 features’ SHAP value in Supplementary Figure S4, which displays an information-dense summary of how the top features in training and independent test datasets impact the PEPPER’s output. Each sample is represented by a single dot on each feature row, and the *×* position is determined by the SHAP value of the feature, while colour in beeswarm plots shows the original value of the feature. We can explore the directionality of each feature from the beeswarm plots. For example, we can find that when ‘GG.gap2′ takes a higher value, PEPPER is more likely to predict the sample as PE_PGRS protein, while when ACC feature ‘I’ takes a higher value, the prediction result is less likely to be positive.

Overall, several important observations can be explored from Fig. 4**D, S2, and S3**. First, we can find three CKSAAP features (‘GG.gap2′, ‘GG.gap4′, and ‘GG.gap5′) and two AAC features (‘G’ and ‘I’) in the top five important features. The three CKSAAP features are all for amino acid (AA) pair GG, and ‘gap2′, ‘gap4′, and ‘gap5′ represent the AA pair separated by 2, 4, and 5 residues, respectively. These results are consistent with the findings of sequence analysis that PE_PGRS proteins are glycine (G)-rich proteins, and several sequence motifs contained glycines (G), such as GGA, GGN, and GGAGGX. Second, the physicochemical property features, such as hydrophobicity, charge, and solvent accessibility from the CTD feature group are also very important for PEPPER. Third, most features’ higher value is more likely to predict the sample as a positive one, while several features are opposite. Altogether, these results demonstrate that both sequence profiles and physicochemical properties contributed to the outstanding predictive performance of PEPPER.

### 3.7 Case studies

**Fig. 5:**
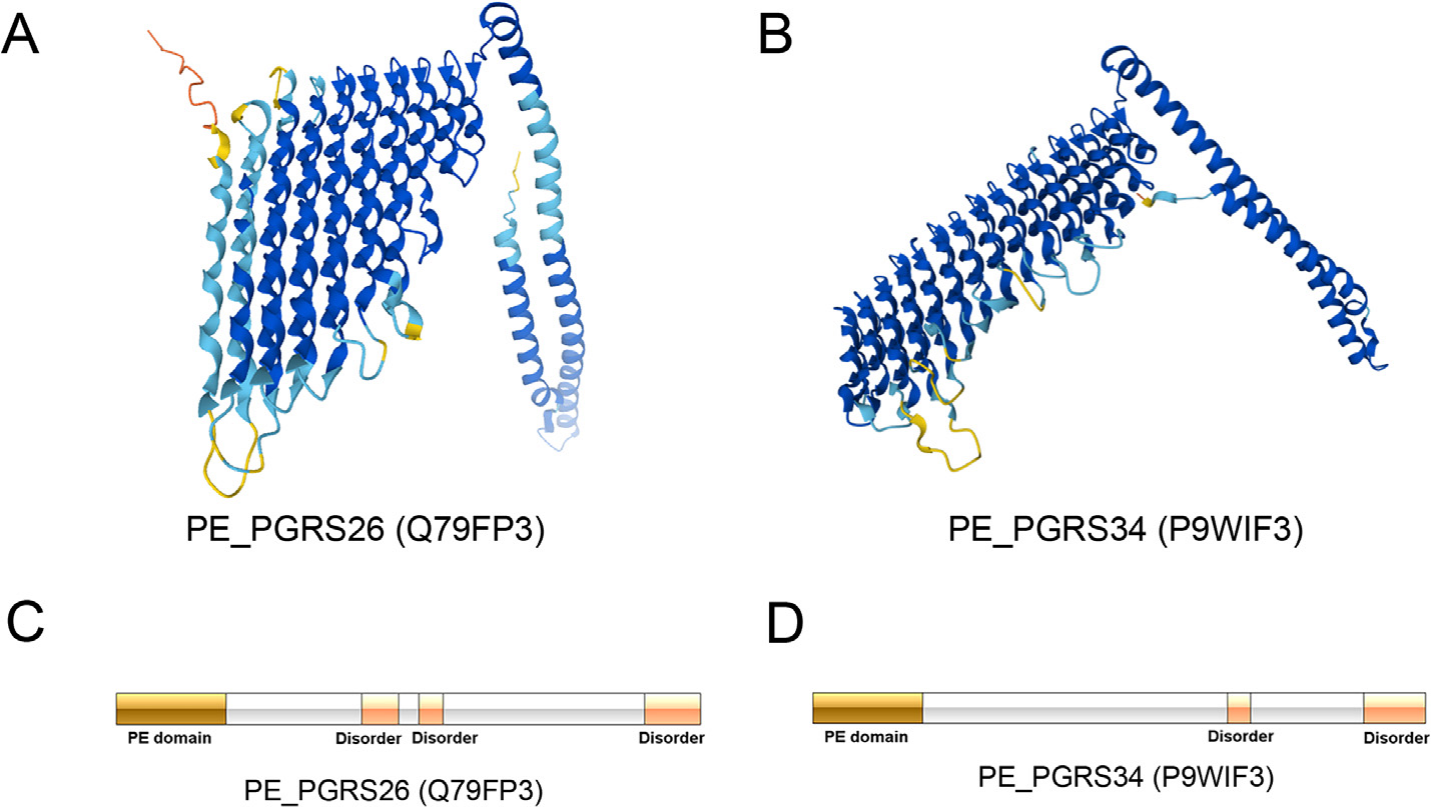
(**A**) Protein 3D structure of PE_PGRS26 (UniProt Accession: Q79FP3) predicted by AlphaFold2. (**B**) Protein 3D structure of PE_PGRS34 (UniProt Accession: P9WIF3) predicted by AlphaFold2. (**C**) Domain and disorder regions of two case study proteins. (**D**) Visualisation of the enriched Gene Ontology terms for the predicted PE_PGRS proteins.

To further illustrate the capacity of PEPPER, we performed case studies of two PE_PGRS proteins from the independent test dataset. PEPPER can successfully predict these two proteins as PE_PGRS proteins while BLASTP and PHMMER cannot with default parameters. The first protein is PE-PGRS family protein PE_PGRS26 (Gene: PE_PGRS26; UniProt ID: PG26_MYCTU; UniProt Accession: Q79FP3), and the second protein is PE-PGRS family protein PE_PGRS34 (Gene: PE_PGRS34; UniProt ID: PG34_MYCTU; UniProt Accession: P9WIF3). We predicted the protein 3D structure of these two case study proteins using AlphaFold2 and visualised them in Fig. 5**A and 5B**. We can find that the structures of these two proteins are very similar, and they are primarily composed of alpha-helices. This is because glycine is integral to form alpha-helices and PE_PGRS proteins are glycine enriched. The domain and disordered regions of these two proteins are presented in Fig. 5**C**, and we can find they both have a PE domain at the N-terminal and ended with a disorder region at the C-terminal.

### 3.8 Proteome-wide prediction and gene ontology enrichment analysis

In this section, we applied PEPPER to pre-compute a comprehensive proteome-wide prediction of PE_PGRS proteins for Mycobacterium. We collected 190,061 *Mycobacterium* proteins from the TrEMBL database, which is the automatically annotated and not reviewed database in the UniProt database. To obtain high-confidence prediction results, we also applied the probability threshold at 99.99%, 99%, and 80% to conduct the prediction. The statistical summary of the predicted PE_PGRS proteins with the probability thresholds 50%, 80%, 99%, and 99.99% is provided in Table 4. A complete list of the predicted PE_PGRS proteins at these four thresholds are freely available at the download webpage of the PEPPER webserver.

**Table 4:** Statistical summary of the proteome-wide prediction of PE_PGRS proteins at 50%, 80%, 99%, and 99.99%

Probability threshold	Number of predicted PE_PGRS proteins
99.99%	1,939
99%	6,584
80%	10,216
50%	13,596

### 3.9 Webserver/software development

In order to facilitate community-wide efforts in performing high-throughput analysis and prediction of novel PE_PGRS proteins, we developed a local stand-alone tool and an online webserver for PEPPER, which are freely available at http://web.unimelb-bioinfortools.cloud.edu.au/PEPPER/. The local stand-alone tool was developed with Python, and the web page of PEPPER was developed based on PHP and managed by Apache HTTP Server and configured in an 8-core Linux server machine with 32 GB RAM and 500 GB hard disk supported by the Melbourne Research Cloud of The University of Melbourne. Users can input their amino acid sequences of interest or upload an input sequence file in the FASTA format, then the task will be submitted to the server-side to make the prediction, and the results will return to the webpage or email to the user’s optionally provided email address. A detailed step-by-step user manual for using the PEPPER web server can be found on the help page of the webserver. Besides, the local stand-alone tool is provided on the website, and users can download it to conduct large-scale high-throughput predictions.

In addition, to demonstrate the computational efficiency of PEPPER, we conducted a performance comparison of PEPPER with BLASTP and PHMMER by using the independent test dataset on the server machine of PEPPER webserver (8-core Linux server machine with 32 GB RAM and 500 GB hard disk). We conducted 10 times experiments and reported the average time used for predicting PE_PGRS proteins. The time-usage comparison results are provided in Table 5. The results show that BLASTP and PHMMER required considerable computational times compared with PEPPER, which is 350 times and 400 times longer than PEPPER, respectively. Therefore, PEPPER significantly reduces the calculation time compared with two alignment-based approaches and three remote homology detection tools, and provides a high-throughput prediction ability for PE_PGRS proteins.

**Table 5:** Time usage comparison results between PEPPER, BLASTP, PHHMER, ProDec-BLSTM, ProDet-CCH, and HHsuite for PE_PGRS protein prediction on the independent test dataset.

Approaches	Average time usage
PEPPER	0 min 24.777 s
BLASTP	145 min 13.675 s
PHMMER	167 min 7.273 s
ProDec-BLSTM	115 min 31.8 s
ProDet-CCH	251 min 50.2 s
HHsuite	248 min 13.8 s

### 3.10 Limitations and future work

Despite the performance of PEPPER for predicting PE_PGRS proteins in *Mycobacterium*, it has the following limitations.

The first limitation is that PEPPER is a machine learning-based approach trained on multiple manually designed sequence-derived features. As is widely known, the effectiveness of machine learning models depends largely on the feature representations used for training. This study only considered sequence profile and amino acid physicochemical property features. However, features from other perspectives, such as protein 3D structural features, can help further improve the prediction performance and enhance the understanding of the 3D structural preferences of PE_PGRS proteins. Therefore, in the future, we plan to map the protein sequence to the 3D structures and explore the 3D structural preference of PE_PGRS proteins.

The second limitation is that PEPPER just focused on PE_PGRS proteins, which is a subfamily of the PE family. PEPPER can only be used as a touchstone to help explore the properties of a small part of this complex protein family. Consequently, we plan to develop a comprehensive database and machine learning-based model to systematically explore the characteristics of the whole PE family proteins in the future.

## 4 Conclusion

This study developed the first machine learning-based predictor, PEPPER, which can identify PE_PGRS proteins rapidly and accurately compared with conventional alignment-based approaches BLASTP and PHMMER. To find the optimal machine learning algorithm to build the classifier, we conducted a comprehensive performance evaluation of 13 popular machine learning algorithms combined with three groups of sequence and physicochemical features for predicting PE_PGRS proteins. In addition, two types of feature selection strategies were evaluated and employed to select the optimal features to further improve the predictive performance. Consequently, PEPPER was constructed based on an optimised lightGBM model. The empirical studies illustrate PEPPER achieved superior predictive performance and significantly reduces the computational cost compared with two state-of-the-art alignment-based approaches BLASTP and PHMMER. The successful performance of PEPPER can be attributed to four major factors: i) A comprehensive database collected from NCBI and Swiss-Prot databases provides up-to-date knowledge of PE_PGRS proteins; ii) A variety of sequence and physicochemical features provide a better characterisation of PE_PGRS proteins than that of alignment-based approaches; iii) The boost-based ensemble algorithm lightGBM not only reduced the training time but also provided a robust predictive power; iv) The two-step feature selection strategy further improved the model performance and reduced the computational complexity, and the selected features’ importance and contribution were examined by SHAP algorithm. Furthermore, we developed the PEPPER webserver and local stand-alone tool and made them freely available at http://web.unimelb-bioinfortools.cloud.edu.au/PEPPER/. We anticipate PEPPER will serve as a valuable tool for facilitating the community-wide efforts for PE_PGRS data analysis. We intend to apply machine learning techniques to develop a prediction system for the whole PE family proteins.

## Declaration of Competing Interest

The authors declare that they have no known competing financial interests or personal relationships that could have appeared to influence the work reported in this paper.
