# Supplementary material for: Computational analysis and prediction of PE_PGRS proteins using machine learning
Source: Comput Struct Biotechnol J. 2022 Jan 22;20:662–74. doi: 10.1016/j.csbj.2022.01.019 (PMC8804200; doi:10.1016/j.csbj.2022.01.019)
Supplement: Supplementary data 1 [file mmc1.docx]

**Supplementary Material**

**Contents**

**Figures:**

[**Figure S1.** Learning curves of PEPPER 2](#_Toc89943840)

**[Figure S2.](#_Toc89943841)** [ROC curves and confusion matrix of the final model on the independent test dataset. 2](#_Toc89943841)

[**Figure S3.** Heatmap plot of the SHAP values for the top 20 important features on the training dataset. 3](#_Toc89943842)

[**Figure S4.** Beeswarm plot of the SHAP values for the top 20 important features on the **(A)** training and (**B**) independent test dataset. 4](#_Toc89943843)

**Tables:**

[**Table S1.** Five groups of amino acids are used for GAAC and CKSAAGP 4](#_Toc89878560)

[**Table S2.** Amino acid physicochemical properties and classification of the amino acids into three groups according to each property 5](#_Toc89878561)

[**Table S3.** Unsupervised analysis results of each feature group and all features 7](#_Toc89878562)

[**Table S4.** Performance comparison results of two feature selection strategies 7](#_Toc89878563)

[**Table S5.** Statistic summarises of 111 selected features through the two-step feature selection (Strategy 2) 7](#_Toc89878564)


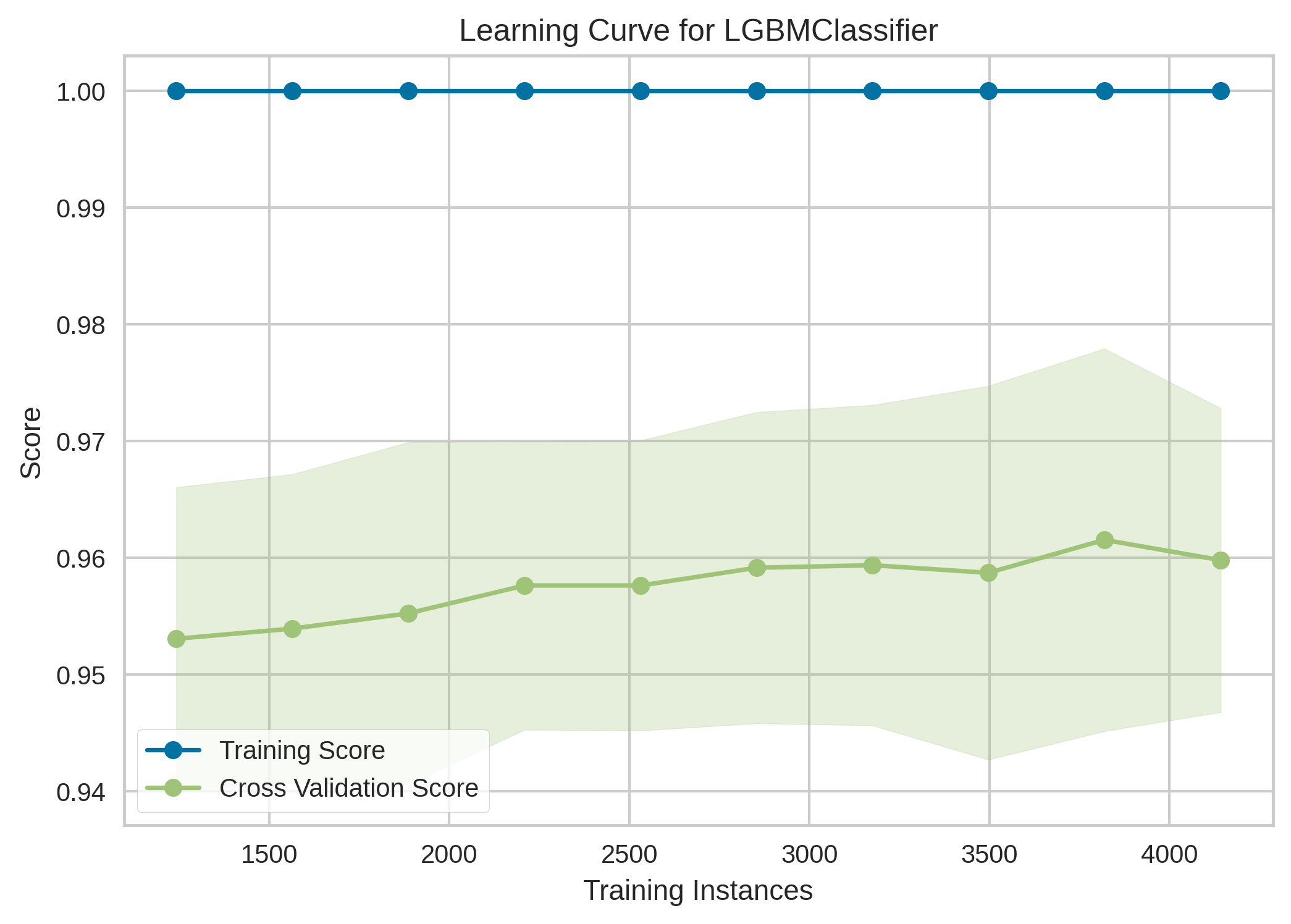


**Figure S1.** Learning curves of PEPPER


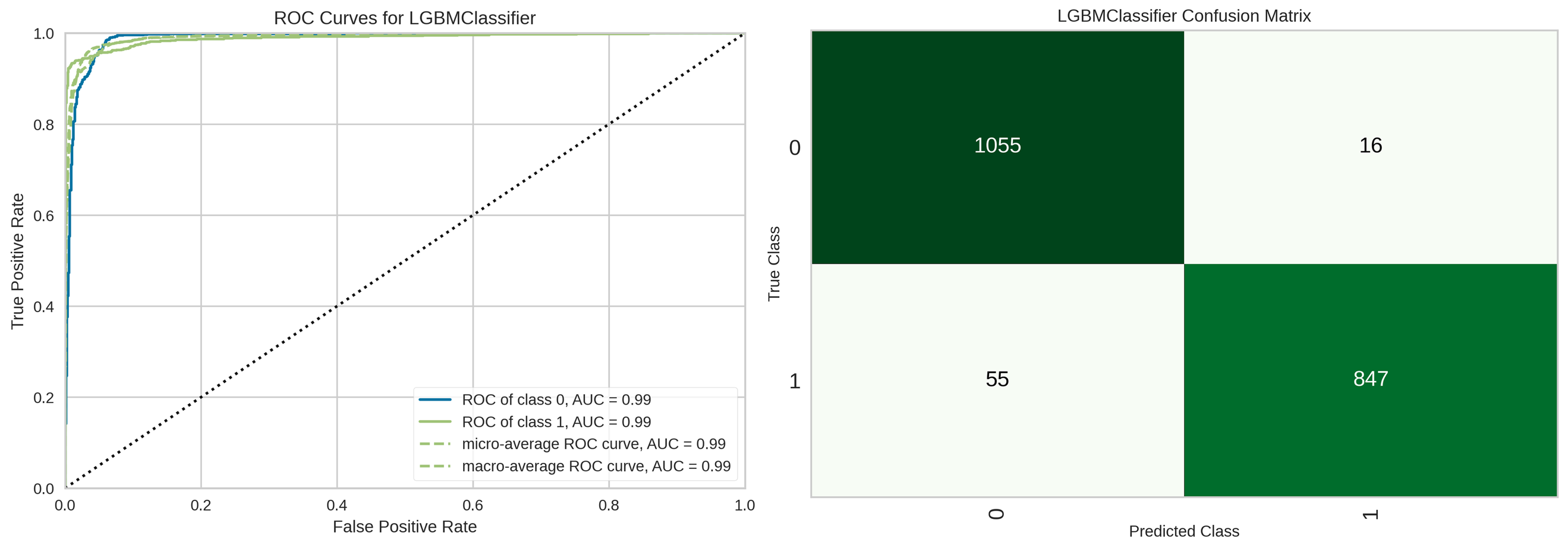


**Figure S2.** ROC curves and confusion matrix of the final model on the independent test dataset.


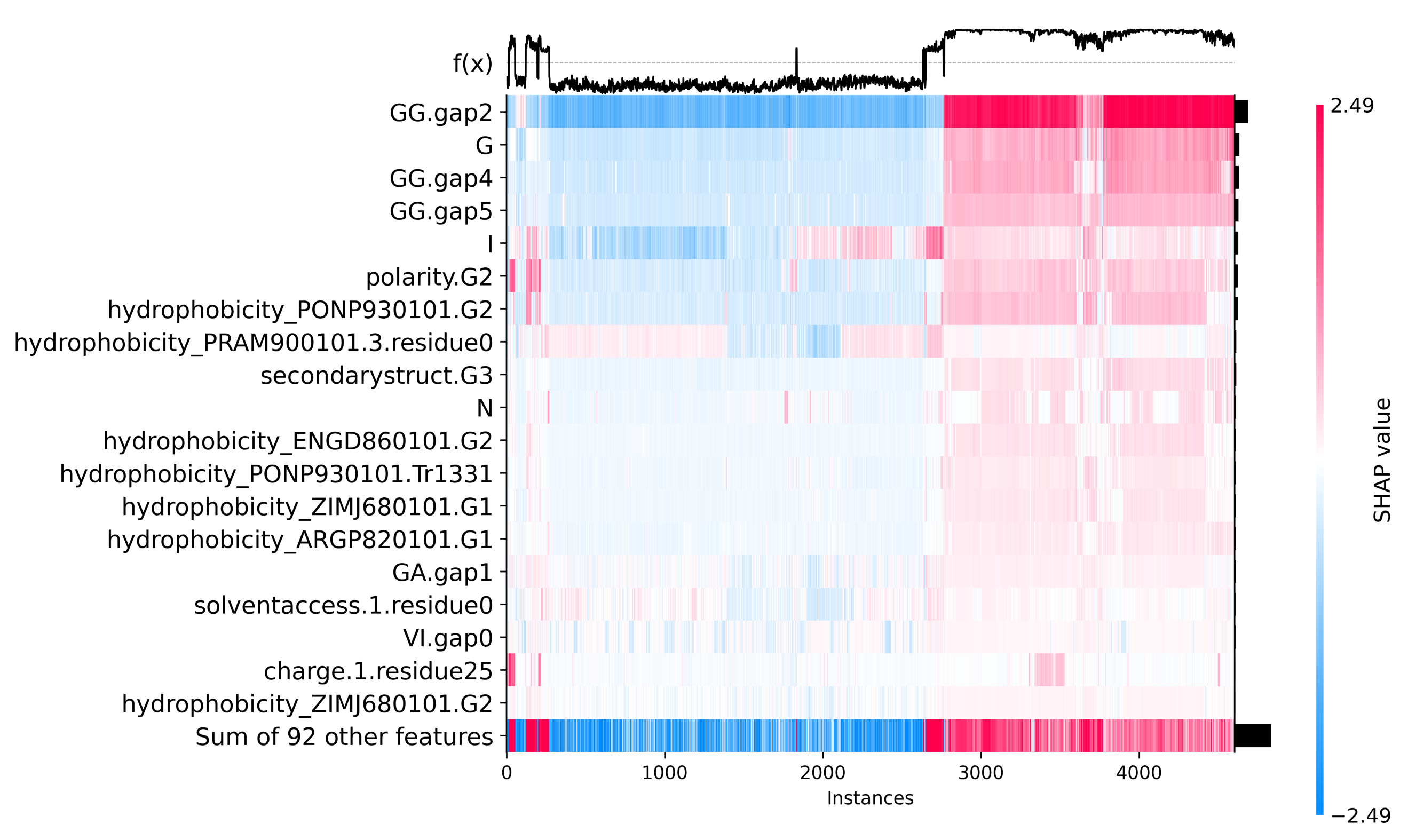


**Figure S3.** Heatmap plot of the SHAP values for the top 20 important features on the training dataset.


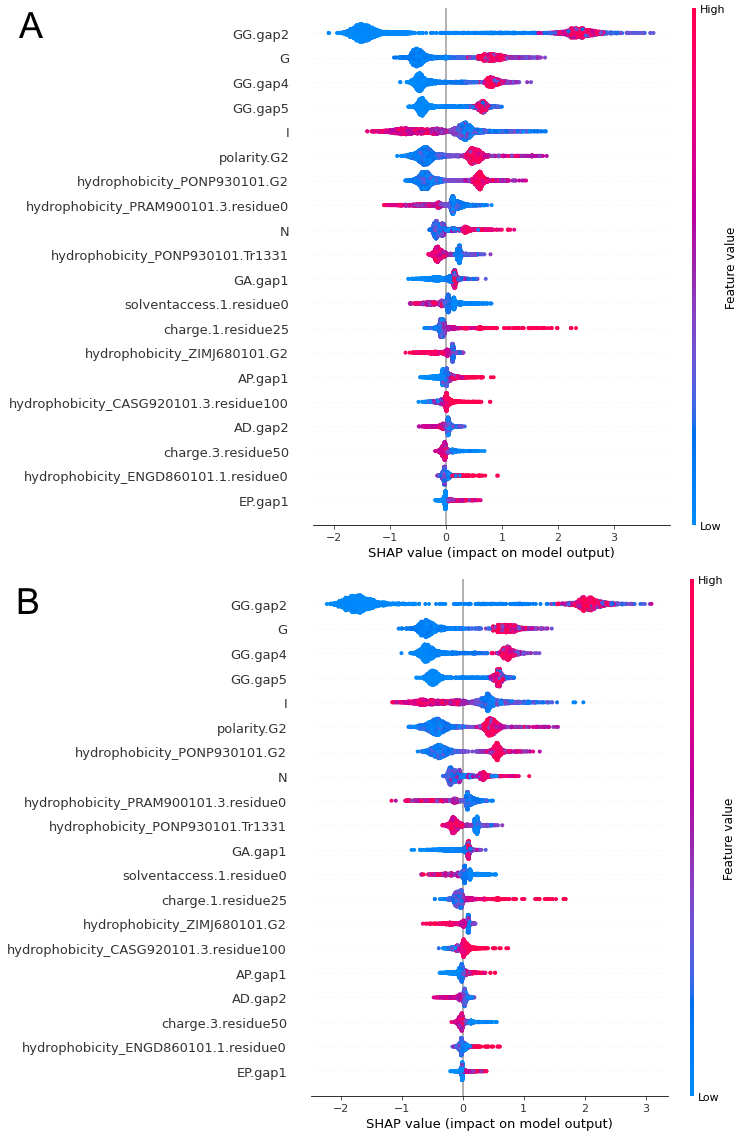


**Figure S4.** Beeswarm plot of the SHAP values for the top 20 important features on the **(A)** training and (**B**) independent test dataset.

**Table S1.** Five groups of amino acids are used for GAAC and CKSAAGP

| **Group** | **Amino acids** |
| --- | --- |
| aliphatic group ($g1$) | G, A, V, L, M, I |
| aromatic group ($g2$) | F, Y, W |
| positive charged group ($g3$) | K, R, H |
| negative charged group ($g4$) | D, E |
| uncharged group ($g5$) | S, T, C, P, N, Q |

**Table S2.** Amino acid physicochemical properties and classification of the amino acids into three groups according to each property

| **Num** | **Properties** | **Group 1** | **Group 2** | **Group3** |
| --- | --- | --- | --- | --- |
| 1 | Hydrophobicity_PRAM900101 | Polar: R; K; E; D; Q; N | Neutral: G; A; S; T; P; H; Y | Hydrophobicity: C; L; V; I; M; F; W |
| 2 | Hydrophobicity_ARGP820101 | Polar: Q; S; T; N; G; D; E | Neutral: R; A; H; C; K; M; V | Hydrophobicity: L; Y; P; F; I; W |
| 3 | Hydrophobicity_ZIMJ680101 | Polar: Q; N; G; S; W; T; D; E; R; A | Neutral: H; M; C; K; V | Hydrophobicity: L; P; F; Y; I |
| 4 | Hydrophobicity_PONP930101 | Polar: K; P; D; E; S; N; Q; T | Neutral: G; R; H; A | Hydrophobicity: Y; M; F; W; L; C; V; I |
| 5 | Hydrophobicity_CASG920101 | Polar: K; D; E; Q; P; S; R; N; T; G | Neutral: A; H; Y; M; L; V | Hydrophobicity: F; I; W; C |
| 6 | Hydrophobicity_ENGD860101 | Polar: R; D; K; E; N; Q; H; Y; P | Neutral :S; G; T; A; W | Hydrophobicity: C; V; L; I; M; F |
| 7 | Hydrophobicity_FASG890101 | Polar: K; E; R; S; Q; D | Neutral: N; T; P; G | Hydrophobicity: A; Y; H; W; V; M; F; L; I; C |
| 8 | Normalised van der Waals volume | 0-2.78: G; A; S; T; P; D; C | 2.95-4.0: N; V; E; Q; I; L | 4.03-8.08: M; H; K; F; R; Y; W |
| 9 | Polarity | 4.9-6.2: L; I; F; W; C; M; V; Y | 8.0-9.2: P; A; T; G; S | 10.4-13.0: H; Q; R; K; N; E; D |
| 10 | Polarizability | 0-0.108: G; A; S; D; T | 0.128-0.186: C; P; N; V; E; Q; I; L | 0.219-0.409: K; M; H; F; R; Y; W |
| 11 | Charge | Positive: K; R | Neutral: A; N; C; Q; G; H; I; L; M; F; P; | Negative: D; E |
| 12 | Secondary Structure | Helix: E; A; L; M; Q; K; R; H | Strand: V; I; Y; C; W; F; T | Coil: G; N; P; S; D |
| 13 | Solvent Accessibility | Buried: A; L; F; C; G; I; V; W | Exposed: R; K; Q; E; N; D | Intermediate: M; S; P; T; H; Y |

**Table S3.** Unsupervised analysis results of each feature group and all features

| **Feature group** | **Cluster 1** | | **Cluster 2** | |
| --- | --- | --- | --- | --- |
|  | **Num. of positives** | **Num. of negatives** | **Num. of positives** | **Num. of negatives** |
| Group 1 | 880 | 3527 | 2161 | 8 |
| Group 2 | 887 | 3529 | 2154 | 6 |
| Group 3 | 273 | 2610 | 2768 | 925 |
| All features | 2274 | 10 | 767 | 3525 |

**Table S4.** Performance comparison results of two feature selection strategies

|  |  | **Accuracy** | **AUC** | **Recall** | **Precision** | **F1** | **MCC** | **Num of Features** |
| --- | --- | --- | --- | --- | --- | --- | --- | --- |
| Training | Original | 0.9533 | 0.9861 | 0.9168 | 0.9814 | 0.9478 | 0.9076 | 3534 |
|  | Strategy 1 | 0.9563 | 0.9857 | 0.9177 | **0.9874** | 0.9511 | 0.9140 | 3055 |
|  | Strategy 2 | **0.9600** | **0.9911** | **0.9266** | 0.9865 | **0.9555** | **0.9210** | **111** |
| Testing | Original | 0.9620 | 0.9893 | 0.9302 | **0.9859** | 0.9572 | 0.9243 | 3534 |
|  | Strategy 1 | 0.9589 | 0.9882 | 0.9290 | 0.9801 | 0.9539 | 0.9180 | 3055 |
|  | Strategy 2 | **0.9640** | **0.9896** | **0.9390** | 0.9815 | **0.9598** | **0.9280** | **111** |

**Table S5.** Statistic summarises of 111 selected features through the two-step feature selection (Strategy 2)

| **Group** | **Feature type** | **Num. of selected features** | **Selected features** |
| --- | --- | --- | --- |
| Group 1 | AAC | 6/20 (30%) | I; N; G; D; L; K |
|  | GAAC | 0/5 (0%) | - |
|  | CKSAAP | 62/2400 (2.58%) | GG.gap2; GA.gap1; GG.gap5; GG.gap4; AP.gap1; AD.gap2; EP.gap1; VI.gap0; EI.gap5; RP.gap0; IG.gap3. AM.gap1; VT.gap0; TR.gap1; RR.gap4; QD.gap5; AP.gap0; MT.gap0; LV.gap1; PR.gap2; IG.gap5; TY.gap4; TV.gap2; PR.gap0; LI.gap3; SF.gap0; IT.gap0; AF.gap3; LA.gap3; HL.gap4; GI.gap5; EG.gap0; EE.gap0; WH.gap0; AA.gap0; LA.gap2; LD.gap1; IV.gap1; EL.gap0; HI.gap5; FL.gap0; VK.gap1; YI.gap2; YL.gap0; VP.gap2; PA.gap2; IK.gap3; ID.gap0; IR.gap5; PR.gap5; GY.gap5; EG.gap1; LV.gap0; LT.gap3; SP.gap0; RH.gap4; ST.gap5; DV.gap0; SR.gap1; KN.gap3; SS.gap5; RD.gap0 |
|  | CKSAAGP | 6/150 (4%) | postivecharger.alphaticr.gap1; alphaticr.uncharger.gap4; uncharger.uncharger.gap5; alphaticr.postivecharger.gap5; negativecharger.alphaticr.gap1; negativecharger.aromatic.gap4 |
| Group 2 | CTDC | 12/39 (30.77%) | polarity.G2; hydrophobicity_PONP930101.G2; hydrophobicity_ZIMJ680101.G2; hydrophobicity_ARGP820101.G1; hydrophobicity_ZIMJ680101.G1; hydrophobicity_PRAM900101.G2; secondarystruct.G3; hydrophobicity_ENGD860101.G2; hydrophobicity_ENGD860101.G3; hydrophobicity_PRAM900101.G3; hydrophobicity_PONP930101.G3; secondarystruct.G2 |
|  | CTDT | 5/39 (12.82%) | hydrophobicity_PONP930101.Tr1331; polarity.Tr1331; hydrophobicity_FASG890101.Tr1331; hydrophobicity_FASG890101.Tr1221; charge.Tr2332 |
|  | CTDD | 15/195 (7.69%) | solventaccess.1.residue0; charge.1.residue25; hydrophobicity_PRAM900101.3.residue0; charge.3.residue50; hydrophobicity_ENGD860101.1.residue0; hydrophobicity_CASG920101.3.residue100; normwaalsvolume.2.residue0; hydrophobicity_ENGD860101.1.residue50; charge.3.residue25; polarizability.2.residue0; hydrophobicity_PONP930101.2.residue0; charge.1.residue0; hydrophobicity_CASG920101.3.residue0; solventaccess.3.residue100; hydrophobicity_FASG890101.1.residue0 |
| Group 3 | CTriad | 5/343 (1.46%) | g3.g4.g3; g1.g2.g3; g3.g1.g3; g6.g1.g5; g5.g4.g6 |
|  | KSCTriad | 0/343 (0%) | - |
